# Good Rules for Winter

**Published:** 1888-02

**Authors:** 


### GOOD RULES FOR WINTER.

Never lean with the back upon anything that is cold.

Never begin a journey until the breakfast has been eaten.

Never take warm drinks and then immediately go out into the cold.

Keep the back especially between the shoulder blades, well covered; also, the chest well protected. In sleeping in a cold room establish a habit of breathing through the nose, and never with the mouth open.

Never go to bed with cold or damp feet

Never omit regular bathing, for unless the skin is in active condition, the cold will close the pores and favor congestion and other diseases.

After exercise of any kind, never ride in an open carriage or near the window of a car for a moment; it is dangerous to health or even life.

When hoarse, speak as little as possible until the hoarseness is recovered from, else the voice may be permanently lost, or difficulties of the throat be produced.

Merely warm the back by the fire, and never continue keeping the back exposed to heat after it has become comfortably warm. To do otherwise is debilitating.

When going from a warm atmosphere into a cooler one, keep the mouth closed so that the air may be warmed in its passage through the nose ere it reaches the lungs.

Never stand still in cold weather, especially after having taken a slight degree of exercise, and always avoid standing on ice or snow, or where the person is exposed to cold wind.—*Sanitarium.*

